# Cross-breed comparisons identified a critical 591-kb region for bovine carcass weight QTL (*CW-2*) on chromosome 6 and the Ile-442-Met substitution in *NCAPG *as a positional candidate

**DOI:** 10.1186/1471-2156-10-43

**Published:** 2009-08-04

**Authors:** Kouji Setoguchi, Masako Furuta, Takashi Hirano, Tomoko Nagao, Toshio Watanabe, Yoshikazu Sugimoto, Akiko Takasuga

**Affiliations:** 1 Cattle Breeding Development Institute of Kagoshima Prefecture, Osumi, So, Kagoshima 899-8212, Japan; 2 Kumamoto Prefectural Agricultural Research Center, Sakae, Koushi, Kumamoto 861-1113, Japan; 3 Shirakawa Institute of Animal Genetics, Japan Livestock Technology Association, Odakura, Nishigo, Fukushima 961-8061, Japan

## Abstract

**Background:**

Growth-related traits have been mapped on bovine chromosome 6 (BTA 6) in various bovine breed populations. We previously mapped a significant quantitative trait locus (QTL) for carcass and body weight (*CW-2*) between 38 and 55 cM on BTA 6 using a Japanese Black half-sib family. Additional QTL mapping studies detected four QTL for body or carcass weight that overlapped with *CW-2 *in Japanese Black and Japanese Brown half-sib families. To map the region in greater detail, we applied cross-breed comparisons of haplotypes that have been shown to be powerful in canine.

**Results:**

We used 38 microsatellite markers to search for a shared *Q *(increasing carcass and/or body weight) haplotype within the 17-cM *CW-2 *region among five sires. Linkage disequilibrium mapping using maternal alleles of the offspring showed that an 815-kb shared *Q *haplotype was associated with body or carcass weight in both breeds. The addition of 43 single nucleotide polymorphism (SNP) markers narrowed the region to 591 kb containing 4 genes. The SNP changing Ile-442 to Met in *NCAPG *(chromosome condensation protein G) was significantly associated with carcass weight (*p *< 1.2 × 10^-11^) in a large Japanese Black population as well as in the five families. The *Q *allele of the SNP was also associated with a larger longissimus muscle area and thinner subcutaneous fat thickness in steers of all five families, indicating that the *CW-2 *locus is pleiotropic and favorable for marker-assisted selection of beef cattle.

**Conclusion:**

A 591-kb critical region for *CW-2 *was identified. The SNP changing Ile-442 to Met in *NCAPG *(chromosome condensation protein G) can be used as a positional candidate of *CW-2 *for marker-assisted selection.

## Background

Body size is one of the most visible animal characteristics and many genes can affect body size. In cattle, body size is correlated with meat quantity, an economically important trait that varies within and across breeds. We previously performed bovine quantitative trait locus (QTL) mapping for growth and carcass traits using Japanese Black paternal half-sib families constructed from a commercial population [1-3]. In these studies, a carcass weight QTL, *CW-1 *on bovine chromosome 14 (BTA 14), was detected in five families with significant linkages and successfully narrowed down to a 1.1-Mb region by identical-by-descent mapping [3] and linkage disequilibrium (LD) mapping [4]. In contrast with *CW-1*, another significant QTL for carcass and body weight, designated *CW-2*, on BTA 6 was replicated at a 1% chromosome-wise significance level, but the two significantly segregating sires had no apparent shared *Q *haplotypes [3]. LD mapping only narrowed the region down to 13.1 Mb (see Results). We thus changed strategies to narrow down the *CW-2 *region. We recently detected carcass weight QTL in regions that overlapped with *CW-2 *in Japanese Brown populations. The Japanese Black breed was established in 1948, basically from indigenous populations of the Japanese Islands, while the Japanese Brown breed originated from indigenous populations of the Korean Peninsula. Approximately 100 years ago, the Korean cattle were imported to Japan and crossed several times with Simmental bulls, followed by the establishment of the Japanese Brown breed in 1948. Therefore, it can be estimated that the two indigenous populations have survived separately for thousands of years. It will be interesting if a common carcass weight QTL is present across the entire *Bos taurus *population. Indeed, the *CW-2 *region was repeatedly highlighted for its association with growth-related traits in a mixed breed population [5], postweaning growth in a beef cattle population [6], and weight and body length at birth in a Charolais × Holstein cross-bred population [7]. These data indicate that *CW-2 *may be shared across breeds. If this is the case, cross-breed comparisons might be useful for fine-mapping to identify a shared hypothetical identical-by-descent haplotype.

In dogs, cross-breed comparisons have been proposed for fine-mapping to pinpoint disease-related genes [8]. Canine LD patterns reflect two bottlenecks in dog history (early domestication and recent breed creation) and long-range breed-specific haplotype blocks retain the underlying short-range ancestral haplotype blocks, suggesting that genetic risk factors are shared across breeds. This situation provides for an efficient mapping strategy: initial mapping within breeds and subsequent fine-mapping by cross-breed comparisons. The strategy was validated in coat color and hair ridge studies [9], in which cross-breed comparisons successfully refined the location of the coat color locus from 800 kb to 100 kb within the pigmentation-related gene. A recent study in cattle also revealed footprints of ancestral LD at short distances (< 10 kb) and these ancestral blocks are organized into larger blocks of a few hundred kilobases within a breed [10].

Here, we demonstrate that cross-breed comparisons are also efficient for fine-mapping of QTL in cattle. The *CW-2 *locus was narrowed down from a 17-cM region to a 591-kb region shared by the Japanese Black and Japanese Brown breeds. Furthermore, a single nucleotide polymorphism (SNP) changing Ile-442 to Met in *NCAPG *(chromosome condensation protein G) was identified as a positional candidate. The *NCAPG-LCORL *locus was recently mapped as a locus associated with human adult height [11,12]. The findings of the present study indicate that genetic variations in *NCAPG *may affect body size in cattle as well as in humans.

## Results

### Carcass and/or body weight QTL in Japanese Black and Japanese Brown cattle

**Figure 1:**
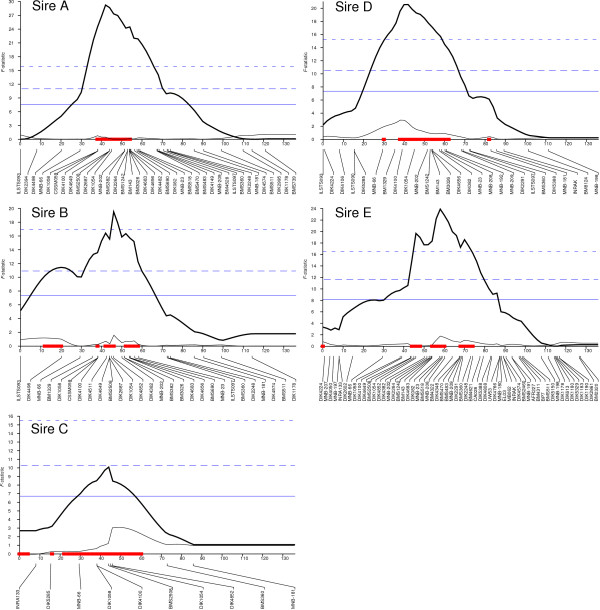
***F*-statistic profiles for carcass or body weight (Sire C only) on BTA 6**. Marker locations were obtained from the Shirakawa-USDA linkage map [20]. Bold and thin lines indicate a fixed-effect QTL model and a fixed-effect QTL model accounting for the effect of SNP-9 of *NCAPG*, respectively. The boxes on the x-axis indicate the 95% CI of the QTL. The horizontal lines indicate the thresholds for chromosome-wise 0.1% (- - -), 1% (- -), and 5% (---) significance levels.

**Figure 2:**
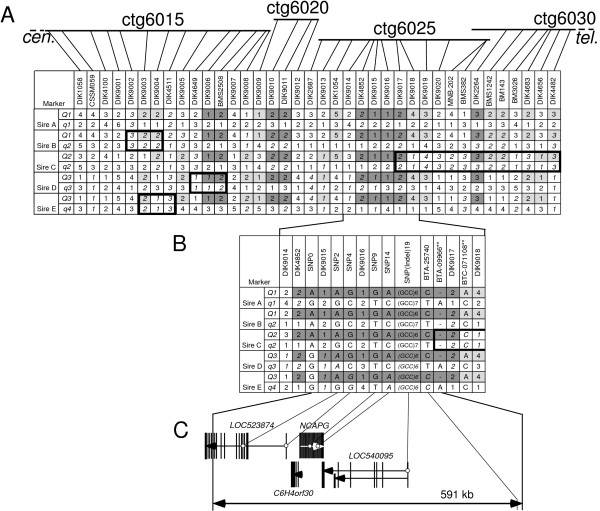
**Haplotype comparison in the *CW-2 *region**. The alleles shared among two or three *Q *haplotypes are colored light grey or grey, respectively. The alleles homozygous in a sire are shown in italics. The regions homozygous for more than two consecutive markers are boxed. (A) Haplotypes between 38 and 55 cM. Locations of the microsatellite markers in the BAC fingerprint map are shown [27]. (B) Haplotypes around the critical 591-kb region. (C) Genes located in the 591-kb region. Exons are indicated by the vertical lines. Each arrow shows the location and direction of a transcript.

Carcass and/or body weight QTL on BTA 6 were mapped in three Japanese Black half-sib families (Sires A-C) and two Japanese Brown half-sib families (Sires D and E; Figure 1; Additional file 1). Sires A and B had the same *Q *haplotype (*Q1*) in the QTL region, whereas Sire C had a different *Q *haplotype (*Q2*) (Figure 2A). Sire E is an offspring of Sire D and inherited the *Q *haplotype of Sire D (*Q3*). In these QTL analyses, the highest *F*-statistic was obtained in Sire A's family, whose QTL was previously designated as *CW-2 *[3]. Because the 95% confidence intervals (CI) of the QTL in the other families overlapped with the *CW-2 *region (38–55 cM), we assumed that these QTL had an identical genetic origin and attempted to find a shared haplotype among the three *Q *haplotypes. By comparing the three *Q *haplotypes using 38 microsatellite markers with an interval of less than 1.18 Mb, three possible identical-by-state (IBS)*Q *regions were detected between 38 and 55 cM: *DIK9006-BMS2508 *(663 kb), *DIK9010–DIK9011 *(537 kb), and *DIK4852–DIK9017 *(815 kb) (Figure 2A; Additional file 2).

### Identical-by-state haplotype associated with carcass and body weight

**Figure 3:**
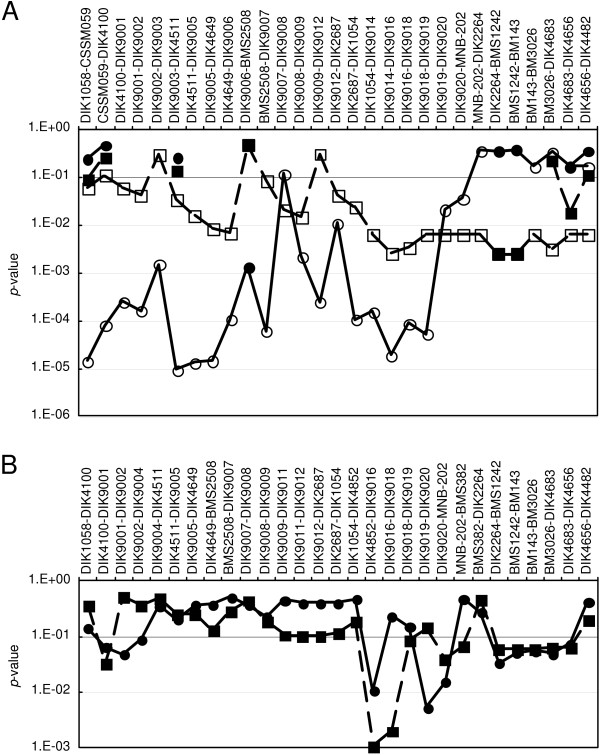
**LD mapping using maternal alleles of the offspring in Japanese Black and Japanese Brown families**. The average of slaughter year- and age-adjusted body or carcass weight was compared between the steers harboring two consecutive *Q *alleles in the maternal alleles and other steers using a *t*-test. The broken and solid lines indicate the *p*-values for the paternal *Q*- and *q*-inherited offspring, respectively. (A) Japanese Black Sire C family. Open and closed marks indicate the *p*-values for the *Q1 *and *Q2 *alleles, respectively. (B) Japanese Brown families of Sires D and E.

To examine whether any of the possible IBS regions were associated with carcass or body weight, we performed LD mapping between 38 and 54 cM using the maternal alleles of the offspring from Sire C (Japanese Black) and Sires D and E (Japanese Brown) (Figure 3). The offspring steers were divided into two groups according to their paternally inherited haplotypes (*Q *or *q*) in this region. The average carcass or body weight was then compared between the steers that harbored two consecutive *Q *alleles in the maternal alleles and the other steers in each group. In Sire C's family, the frequency of two consecutive *Q2 *alleles among the maternal alleles was less than 3% in most regions, while the frequency of two consecutive *Q1 *alleles was greater than 17% between *DIK1058 *and *DIK9019*. Therefore, average body weight was compared between the steers harboring *Q1 *alleles in the maternal alleles and those harboring non-*Q1 *alleles, and between the steers harboring maternal *Q2 *alleles and those harboring non-*Q2 *alleles, in the regions where corresponding *Q *frequencies for two consecutive markers were higher than 5%. Body weight was significantly higher (*p *< 10^-3^) in the regions between *DIK1058 *and *DIK9019 *in the maternal *Q1*-harboring steers than in the non-*Q1-*harboring steers in the paternal *q*-inherited offspring group (Figure 3A). This finding allowed us to narrow down the *CW-2 *region to the 13.1 Mb flanked by *DIK1058 *and *DIK9020*, which contained all possible IBS regions. As for *Q2*, Sire C was homozygous in the telomeric part at the 95% CI (Figures 1 and 2), which allowed us to narrow down the QTL to the centromeric side of *DIK9017*.

In Japanese Brown families, offspring from Sires D and E were combined to maximize the number of offspring. Carcass weight was higher in *DIK4852*-*DIK9016 *(*p *= 0.011) and in *DIK9019*-*MNB202 *(*p *= 0.0052 at *DIK9019–DIK9020*) in the maternal *Q3*-harboring steers than in the non-*Q3-*harboring steers among the offspring with the paternally inherited *q *haplotype (Figure 3B). The two regions were in linkage disequilibrium. Among 161 steers that inherited the paternal *q *haplotype, 50 harbored maternal *Q3 *in *DIK4852*-*DIK9016*, 31 of which harbored maternal *Q3 *in *DIK9019–DIK9020*. On the other hand, all of the steers that harbored maternal *Q3 *in *DIK9019–DIK9020 *also harbored maternal *Q3 *in *DIK4852*-*DIK9016*. The *DIK4852*-*DIK9016 *region was included in the narrowed-down *CW-2 *region and corresponded to one of the possible IBS regions, whereas the *DIK9019*-*MNB202 *region was included in a homozygous region in Sire C. An association of the *DIK4852*-*DIK9016 *region with carcass weight was also observed among offspring with the paternally inherited *Q *haplotype.

These results strongly suggest that the causal alleles in the three QTL haplotypes *Q1*, *Q2*, and *Q3 *are identical, and that the IBS region between *DIK4852 *and *DIK9017 *might be responsible for the QTL.

### Ile-442-Met Mutation as a positional candidate

The possible IBS region between *DIK4852 *and *DIK9017 *corresponds to the 17.2 to 17.9-Mb region on human chromosome 4 that contains six genes (*LAP3, MED28, FAM184B, C6H4orf30, NCAPG*, and *LCORL*) [13-15]. To examine the region in detail, SNPs were searched for by resequencing Sire A in the coding regions of the six orthologous bovine genes (*LAP3, MED28, LOC523874, C6H4orf30, NCAPG*, and *LOC540095*) and genotyped in the five sires (Figure 2B; Additional file 3). This ensured and refined the IBS *Q *region to an 879-kb interval flanked by SNP-0 and BTC-071108**. In addition, the telomeric part of the region from BTA-09966** to BTC-071108** was homozygous in Sire C, resulting in the narrowing of the *CW-2 *region to a 591-kb interval flanked by SNP-0 and BTA-09966** (Figure 2B; Additional file 3).

**Figure 4:**
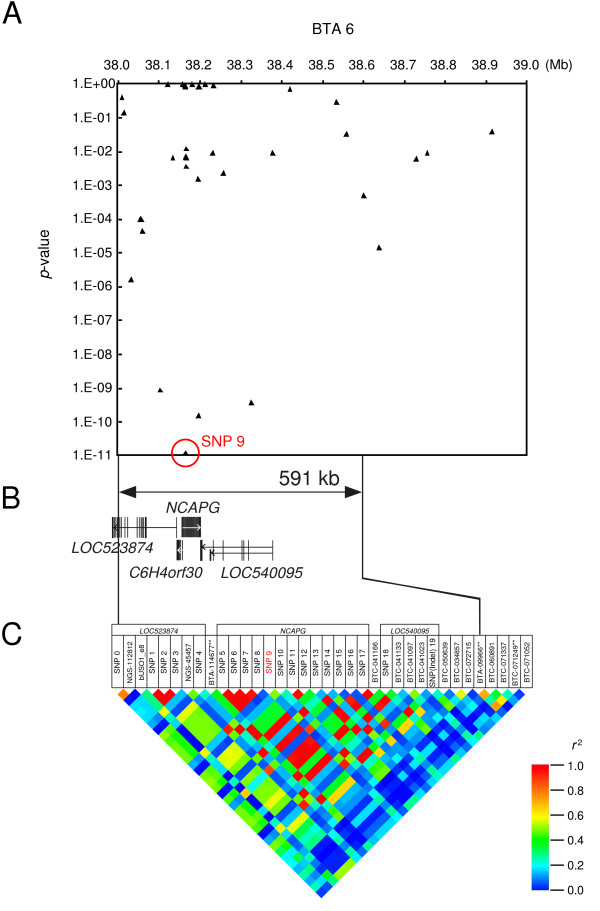
**Association study in the IBS region using a Japanese Black population**. (A) Association of the SNPs with carcass weight. A hundred and eighty-seven steers selected from the 4.7% extremes of 7990 steers were genotyped. *P*-values were calculated by Fisher's exact test for independence. (B) Genes located in the 591-kb region. (C) LD map. The LD was calculated for the 187 steers.

The critical 591-kb region contained four genes (*LOC523874, C6H4orf30, NCAPG*, and *LOC540095*; Figure 2C). In their coding regions, four non-synonymous SNPs and one Indel causing an amino acid insertion were found in Sire A (Figure 2BC; Additional file 3). A causative sequence variation, however, should be heterozygous in all five sires. Among the sequence variations in the coding regions, only the SNP in exon 9 of *NCAPG *(SNP-9) that substitutes a Met (ATG) for Ile-442 (ATT) was heterozygous in all five sires. Four intronic SNPs locating in *LOC523874, NCAPG*, and *LOC540095 *were also heterozygous in all five sires (bUSO1_e8, NGS-45457, SNP16, and BTC-041023 in Figure 4C; Additional file 3).

To assess the association of these SNPs with carcass weight, 187 steers selected from the 4.7% extremes of 7990 Japanese Black steers were genotyped. SNP-9 of *NCAPG *had the strongest association (*p *< 1.2 × 10^-11^, Fisher's exact test for independence; frequency of G-allele, 0.14; Figure 4A; Additional file 3), and it was in strong linkage disequilibrium with the four intronic SNPs (Figure 4BC). These data indicated that SNP-9 is the most appropriate *CW-2 *marker among the examined SNPs as a candidate for the causative sequence variation, although three other genes are in the same LD block and cannot be excluded as candidate genes for *CW-2*.

**Figure 5:**
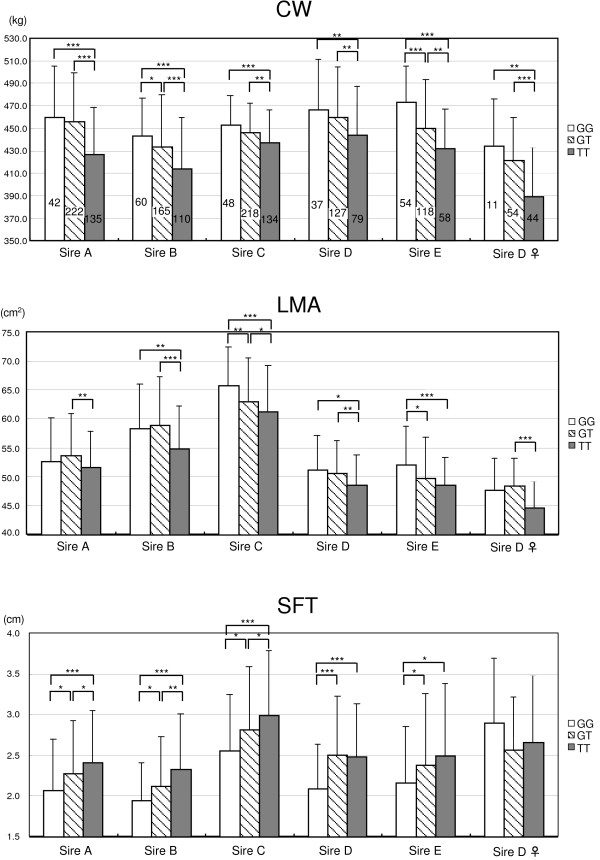
**Association of the SNP-9 genotypes of *NCAPG *with carcass weight, LMA, and SFT**. The steers of the five families and the cows of Sire D's family were genotyped with SNP-9. The number of offspring of each genotype is shown in the bar of the upper panel. The averages of slaughter year- and age-adjusted phenotypic values were compared between genotypes using a *t*-test. *, *p *< 0.05; **, *p *< 0.01; ***, *p *< 0.001.

To further confirm the association of SNP-9 with carcass weight in Japanese Brown as well as Japanese Black populations, the steers of all five families and the cows of Sire D's offspring were genotyped with SNP-9 and average carcass weight was compared between genotypes in each family. Because steers and cows sometimes show different trait characteristics, the cows were separately evaluated from the steers in Sire D family (Figure 5). The G/G-genotype (442-Met/Met of *NCAPG*) and G/T-genotype (442-Met/Ile of *NCAPG*) had significantly greater average carcass weight than the T/T-genotype (442-Ile/Ile of *NCAPG*) in the steers of all five families and the cows of Sire D's offspring (*p *< 0.01, *t*-test). The carcass weight of the G/G-genotype tended to be higher than that of the G/T-genotype, but the difference was significant only in the families of Sires B and E. The effect of SNP-9 was also confirmed by QTL analyses including the genotype of SNP-9 as a fixed effect. The QTL peaks were all extinguished by including the SNP-9 genotype (Figure 1). These data confirmed that SNP-9 of *NCAPG *is a positional candidate for the quantitative trait nucleotide for *CW-2*.

### Pleiotropy of *CW-2*

QTL mapping studies detected QTL for the longissimus muscle area (LMA) and subcutaneous fat thickness (SFT) in the regions overlapping with *CW-2 *at less than 5% chromosome-wise significance levels in families of Sires A and B (Additional file 4). A QTL for LMA was also detected in Sire D's family at the 1% chromosome-wise significance level (Additional file 4). These QTL peaks were also extinguished in a fixed effect QTL model accounting for the effect of SNP-9 (Additional file 4). Therefore the SNP-9 genotypes were tested for LMA and SFT in each family (Figure 5). The G/G- or G/T-genotypes had a significantly greater average LMA than the T/T-genotype in the steers of the five families and the cows of Sire D's offspring (*p *< 0.01, *t*-test), although the correlation between carcass weight and LMA traits was not high (*r *= 0.26–0.54). On the other hand, the G/G-genotype had a significantly lower average SFT than the T/T or G/T-genotypes in the steers of the five families (*p *< 0.039, *t*-test). This is a particularly interesting finding because the correlation between carcass weight and SFT traits was *r *= 0.18–0.39 in each family (Additional file 5), meaning that greater carcass weight was correlated with thicker SFT. For example, a *Q *allele of another carcass weight QTL *CW-1*on BTA 14 is associated with greater carcass weight and thicker SFT [1]. In *CW-2*, however, the SNP-9 genotype was associated with thinner SFT in the steers of the five families, indicating that the *CW-2 *locus is pleiotropic. The results further support the notion that the *CW-2 *QTL in the Japanese Black population is the same as the QTL in a respective position in the Japanese Brown population.

## Discussion

Here we show that cross-breed comparisons are useful for fine-mapping of the QTL in cattle, as recently demonstrated in the fine-mapping of a monogenic trait locus in dogs [9]. We detected a carcass weight QTL on BTA 6 in two breed populations, Japanese Black and Japanese Brown. The genetic distance between the two cattle breeds can be thousands of years, because their ancestors have survived separately on the Japanese Islands and the Korean Peninsula. Linkage disequilibrium mapping using the two breeds narrowed the *CW-2 *QTL region to a 591-kb section. Comparison of the *Q *haplotypes inherited in different lineages is, therefore, a useful strategy for narrowing down a QTL.

One of the critical issues should be whether the *CW-2 Q *in the Japanese Black population is identical to the QTL in the Japanese Brown population. In this study, the effects of the *CW-2 *on carcass weight, LMA, and SFT traits were similar in the two breed populations. Namely, the *CW-2 Q *allele was associated with greater carcass weight, larger LMA, and thinner SFT. Importantly, although greater carcass weight is generally associated with a thicker SFT, in both populations the *CW-2 Q *allele was associated with a thinner SFT. In addition, QTL for growth-related traits were mapped around the *CW-2 *region in various cattle populations [5-7], suggesting that the *CW-2 Q *allele is widely distributed in *Bos taurus *populations.

The 591-kb critical region contains four genes, *LOC523874, C6H4orf30, NCAPG*, and *LOC540095 *(the bovine ortholog of *LCORL*). The *NCAPG-LCORL *region was recently identified as a QTL for human adult height [11,12]. An SNP causing Ile-442 to Met in *NCAPG *was detected as a positional candidate for the quantitative trait nucleotide for *CW-2*. The *NCAPG *gene encodes chromosome condensation subunit G, which is a catalytic subunit of the mammalian condensin I complex. Disruption of the *NCAPG *ortholog (*cnd3*) causes defective chromosome condensation in fission yeast [16], while in Drosophila, the *NCAPG *ortholog (*Cap-G*) is essential for chromosome condensation in the metaphase of single, unreplicated sister chromatids, and also has a role during the interphase in regulating heterochromatic gene expression [17]. In Hela cells, NCAPG interacts with HSF2 to mediate *HSPA1A *(*hsp70i*) bookmarking [18]. The *NCAPG *expression is upregulated in more aggressive metastatic melanomas than in less aggressive primary melanomas [19]. These findings indicate that *NCAPG *may affect cell proliferation and growth through regulating cell cycle and chromosome condensation. Further studies are required to reveal the molecular mechanism that links *NCAPG *to growth and body size.

## Conclusion

We identified a 591-kb critical region for *CW-2*. Among the four genes located within the region, *NCAPG *is a candidate for the causative gene because it has an amino acid substitution that is significantly associated with carcass weight. The SNP for the amino acid substitution provides a useful *CW-2 *marker for marker-assisted selection. The pleiotropic characteristic of *CW-2*, especially the effect on SFT, is clearly different from a previously fine-mapped carcass weight QTL, *CW-1 *[1]. Therefore, the two QTL probably affect carcass weight in different ways and both will be useful for breeding beef cattle.

## Methods

### DNA samples and phenotype data

Paternal half-sib families of Sires A through E were constructed from carcass data and pedigree records collected by the Japan Wagyu Register Association (Kyoto, Japan) and Kumamoto Union of Livestock and Agriculture Cooperation (Kumamoto, Japan). Offspring of each sire were reared in different herds in a prefecture. The offspring of each sire were collected over a period ranging from 2 to 5 years. The average slaughter age was 878 days for the offspring of Japanese Black Sires A through C and 750 days for the offspring of Japanese Brown Sires D and E. Sire DNA was obtained from semen. Offspring DNA samples were collected from adipose tissues around the kidney at the slaughterhouses or blood at the individual farmer's houses. Six traits were analysed in this study: body weight at slaughter (Sire C only), cold carcass weight, LMA, rib thickness (thickness of a muscle layer in a rib of beef), SFT, and marbling. These traits were systematically measured by certified graders and recorded at the slaughterhouses in Japan.

### QTL mapping

The genome or chromosome screen was conducted using the microsatellite markers on the Shirakawa-USDA linkage map [20]. QTL analyses were performed with the interval mapping method using a linear regression model for half-sib families [21,22], as described previously [1]. Linear regression analysis was performed using the following model:



where *y *is the vector of phenotypic value, *X *is the design matrix of fixed effects composed of sex, slaughter year, age (day), and probability of having the *Q *phase at a given location(*Prob(Q)*), *b *is the vector of fixed effects, and *e *is the residual error. *b *was estimated by the least squares method. An *F*-statistic value at each position was calculated from the residual sum square regressed with *Prob(Q)*, and the total residual sum square without *Prob(Q)*. The analysis was performed at 2-cM intervals along each chromosome. To evaluate whether the QTL effect was well estimated, the information content was calculated as a variance of *Prob(Q) *divided by 0.25, which was the maximum possible value of *Prob(Q) *[23]. The allele substitution effect from *q *to *Q *was calculated as an estimator of the cofactor for *Prob(Q) *in the *b*. The contribution ratio was calculated as a proportion of the trait variance explained by the paternal allele substitution from *q *to *Q*. A threshold for significance of the *F*-statistic value was obtained by 10,000 random permutations of the phenotypic data [24]. The 95% CI of the QTL locations was calculated using the bootstrapping method [25]. Briefly, a set of offspring was chosen so as to be the same number as the original half-sibs by resampling from the original half-sibs. Resampling was repeated 10,000 times. The position of the *F*-statistic peak in each bootstrapping was collected. The CI was determined by the distribution of the peaks. Therefore, the CI may be fragmented into separated regions, and not a single contiguous region.

### Microsatellite development and genotyping

Microsatellites were searched for in the genomic sequences and the primers were designed using Primer 3 [26]. Twenty microsatellite markers from *DIK9001 *to *DIK9020 *were developed in this study. The markers were anchored to the BAC fingerprint map [27] by a BLAST search (BLASTN) against bovine BAC sequences (HTGS) [28] or by screening bovine BAC libraries, RPCI-42 [29] and CHORI-240 [30]. Marker information, such as primer sequences, genomic positions, and locations in the BAC fingerprint map, is shown in Additional file 2. Genotyping was performed using polymerase chain reaction (PCR) with a fluorescent-labeled reverse primer, followed by electrophoresis using ABI 3730 DNA analyzer (Applied Biosystems, Foster City, CA) and analysis using GeneMapper software (Applied Biosystems). The sires and their offspring were genotyped to determine the phase of the sires' chromosomes.

### Linkage disequilibrium mapping

Linkage disequilibrium mapping was performed using the maternal alleles of the offspring steers. First, offspring were divided into two groups according to paternally inherited haplotypes (*Q *or *q*) of the *CW-2 *region (38–55 cM). When a crossover was observed or suspected within the region in the paternal haplotype, the offspring were eliminated. The numbers of paternal *Q- *and *q*-inherited steers were 126 and 117 in Sire C's family, 106 and 94 in Sire D's family, and 104 and 67 in Sire E's family, respectively. The maternal allele was determined by subtracting the paternal allele from the genotype. The markers for which the number of alleles was less than four (Sire C family) or three (families of Sires D and E), or heterozygosity was less than 0.4 were eliminated. The effect of pairwise *Q *alleles on body or carcass weight was evaluated in each group using a *t*-test. Phenotypic values for carcass and body weight were adjusted based on slaughter year and age in the family. The average of the adjusted carcass or body weight was compared between the steers that harbored two consecutive *Q *alleles in the maternal alleles and the other steers, in each group. In Sire C family, the test was performed for *Q1 *and *Q2 *alleles in the regions where corresponding *Q *frequencies were higher than 5%. In Japanese Brown families, offspring from Sires D and E were combined to maximize the number of offspring. Carcass weight was not adjusted between families, because the effect of sire was not significant (*p *= 0.21). Frequencies of two consecutive *Q3 *alleles in the maternal alleles were more than 4.3% in the entire region.

### SNP discovery and genotyping

Six genes (*LAP3, MED28, LOC523874, C6H4orf30, NCAPG*, and *LOC540095*) were located between *DIK4852 *and *DIK9017*. SNPs in the coding regions of the genes were searched for by resequencing Sire A. SNPs chosen from a 120 K SNP collection [31] and a commercially available bovine 50 K SNP chip (Illumina, San Diego, CA) were also used. The sires and their offspring were genotyped to determine the phases of the sires' chromosomes. SNPs were genotyped by direct sequencing of the genomic PCR products. As for SNP (Indel) 19, PCR was performed using a fluorescent-labeled reverse primer and genotyped in the same way as a microsatellite marker. SNP information, such as alleles, genomic positions, and primer sequences used for amplification and sequencing, is shown in Additional file 3.

### Association study

A population consisting of 7990 Japanese Black steers was constructed from DNA samples collected at one slaughterhouse over 7 years. These cattle were reared in different herds throughout Japan. To maximize the power and minimize the pedigree effect of a selected population, steers were selected from the 4.7% extremes, from many different sires, and did not include more than 5 half-sibs in each extreme. The selected population consisted of 90 offspring (> 570 kg) from 41 sires and 97 offspring (< 410 kg) from 52 sires, among which 18 sires were common. The 187 steers were genotyped with SNPs. The association of each SNP with carcass weight was assessed by Fisher's exact test for independence using a 2 × 2 contingency table consisting of an observed number of each allele in each extreme population.

### Calculation of LD

SNPs were confirmed not to have deviated from Hardy-Weinberg equilibrium (*p *> 0.05). Haplotype frequencies consisting of two SNPs were estimated using the expectation-maximization algorithm of Dempster et al. [32]. An LD coefficient (*r*^2^) between two SNPs was calculated from the estimated haplotype frequencies.

## Authors' contributions

KS, MF, TH, and TN performed the QTL analyses. KS also performed molecular genetic studies. TW performed the statistical analyses and developed microsatellite markers. YS coordinated the study and helped draft the manuscript. AT designed the study, performed molecular analyses and drafted the manuscript. All authors read and approved the final manuscript.

## Supplementary Material

Additional file 1 **Summary of carcass or body weight QTL detected on BTA 6**. This table shows the summaries of the half-sib QTL analyses. Click here for file

Additional file 2 **Microsatellite marker information**. This table shows primer sequences and physical positions of the microsatellite markers. Click here for file

Additional file 3 **SNPs and haplotypes of the five sires around the IBS region**. This table shows primer sequences and physical positions of the SNP markers and haplotypes of the five sires. Click here for file

Additional file 4 ***F*-statistic profiles for LMA and SFT on BTA 6**. *F*-statistic profiles for LMA and SFT on BTA 6 are shown as Figure 1. Click here for file

Additional file 5 **Correlation between carcass weight and SFT in each family**. Slaughter year- and age-adjusted phenotypic values for carcass weight and subcutaneous fat thickness are plotted by dots colored per genotype. Click here for file
