# Supplementary figures and images for: Cross-breed comparisons identified a critical 591-kb region for bovine carcass weight QTL (CW-2) on chromosome 6 and the Ile-442-Met substitution in NCAPG as a positional candidate
Source: BMC Genet. 2009 Aug 4;10:43. doi: 10.1186/1471-2156-10-43 (PMC2736976; doi:10.1186/1471-2156-10-43)

# LMA

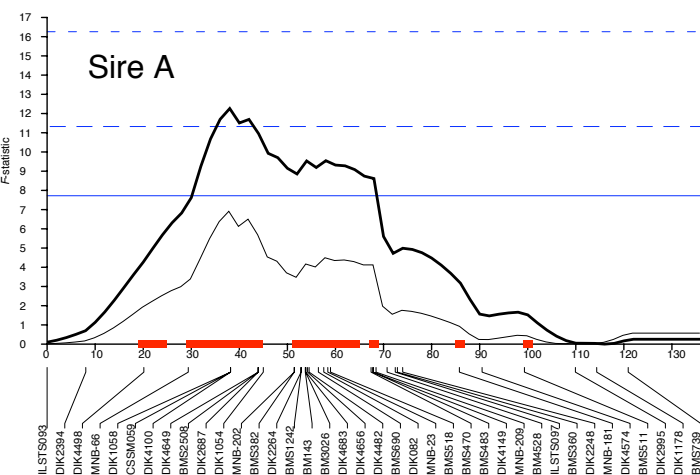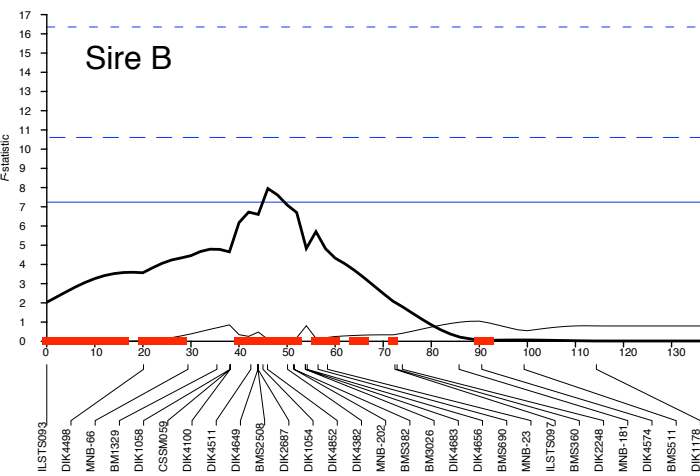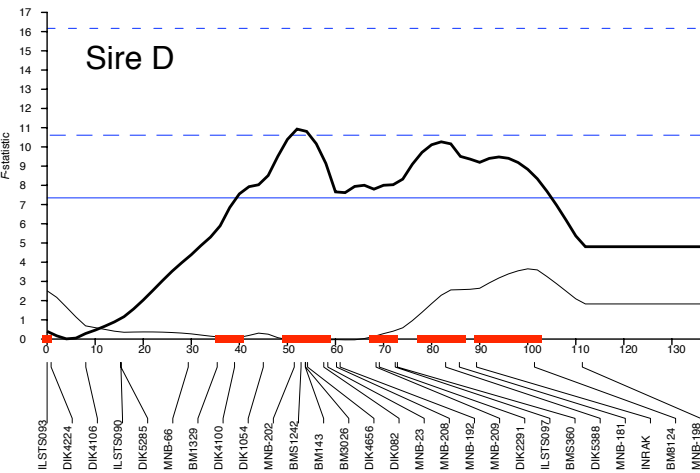

# SFT

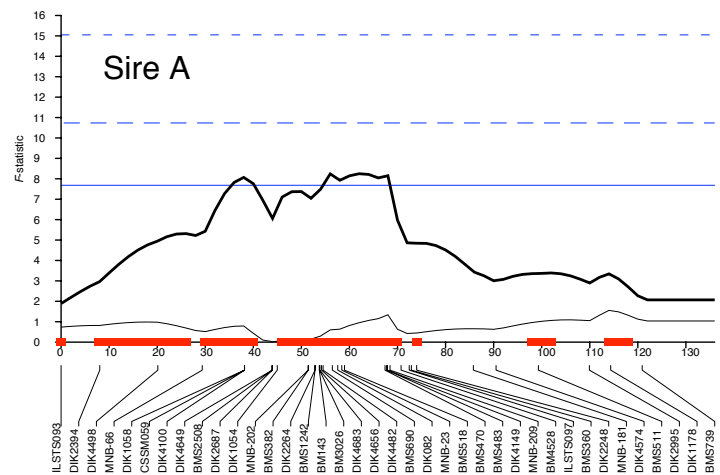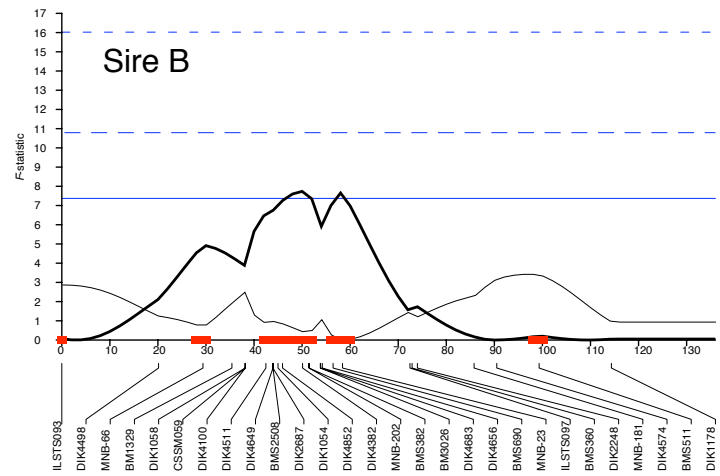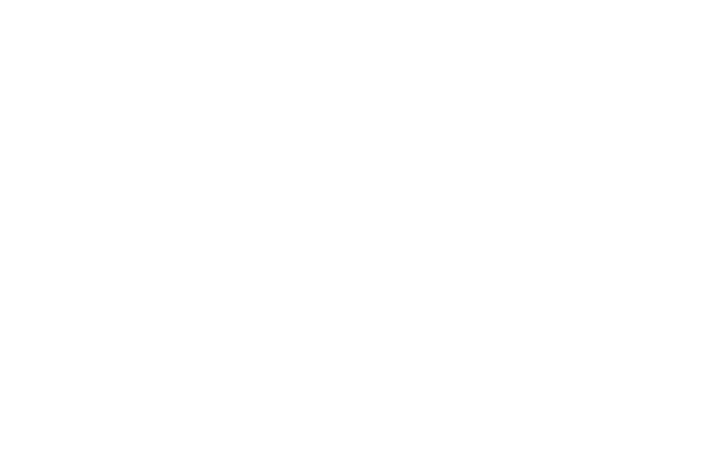

Supplement: Additional file 4 — F-statistic profiles for LMA and SFT on BTA 6. F-statistic profiles for LMA and SFT on BTA 6 are shown as Figure 1. [file 1471-2156-10-43-S4.pdf]

Sire A

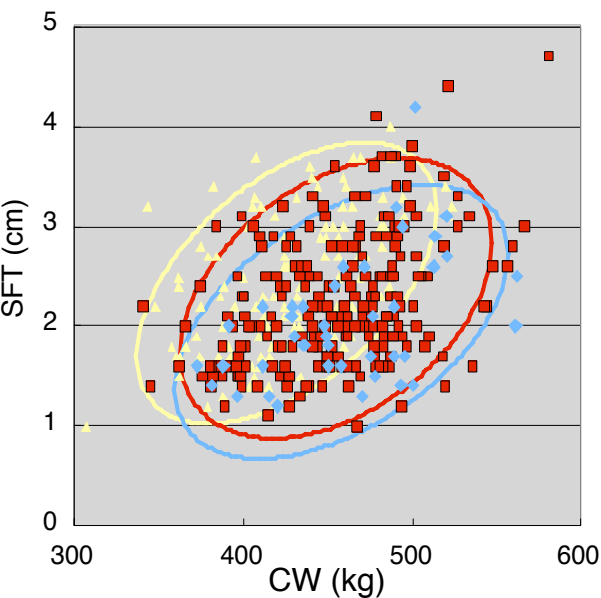

Sire D

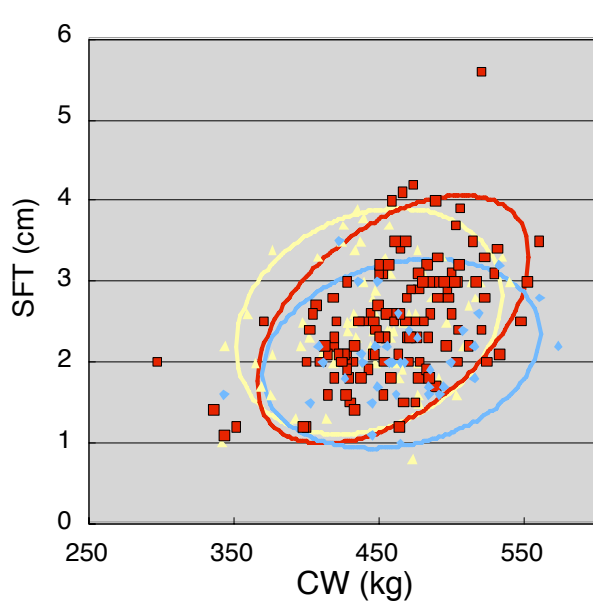

Sire B

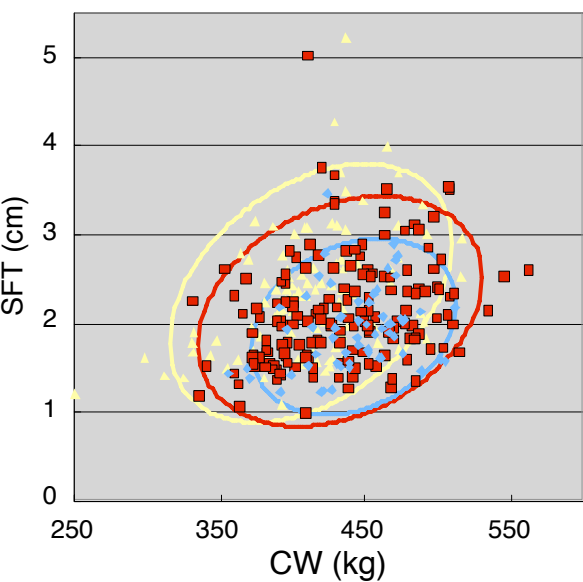

Sire E

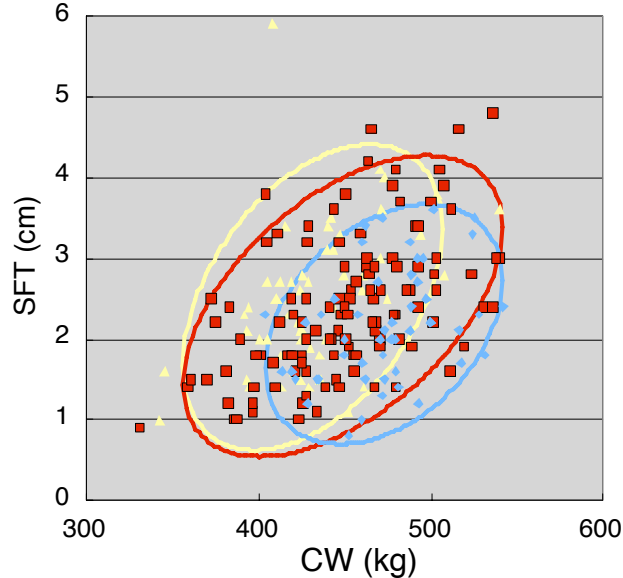

Sire C

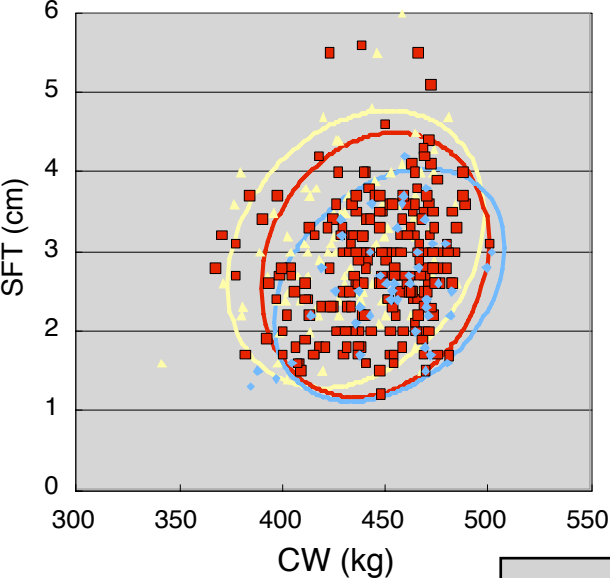

Sire D ♀

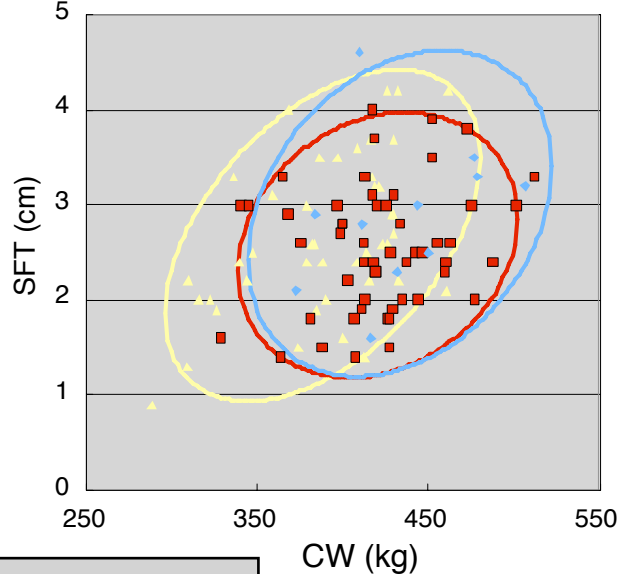

▲ TT, — 90%-CI (TT);  
 ■ GT, — 90%-CI (GT);  
 ◆ GG, — 90%-CI (GG).

Supplement: Additional file 5 — Correlation between carcass weight and SFT in each family. Slaughter year- and age-adjusted phenotypic values for carcass weight and subcutaneous fat thickness are plotted by dots colored per genotype. [file 1471-2156-10-43-S5.pdf]
